# Editorial: Mechanotransduction and development of cardiovascular form and function

**DOI:** 10.3389/fphys.2015.00131

**Published:** 2015-04-28

**Authors:** Kersti K. Linask, Michiko Watanabe

**Affiliations:** ^1^Department of Pediatrics, Morsani College of Medicine, University of South FloridaSt. Petersburg, FL, USA; ^2^Department of Pediatrics, Case Western Reserve UniversityCleveland, OH, USA

**Keywords:** cardiovascular function, congenital heart defects, hemodynamics, mechanotransduction, heart looping, cardiac morphogenesis, placenta, yolk sac

The factors that affect cardiovascular development (molecular, function, and structural changes) are often studied as having a linear relationship with molecular changes being the primary cause of the cascades. It is more likely that these factors are interacting continuously throughout development requiring a more balanced understanding of each factor. We now know substantially about the molecular and structural changes using several different approaches and advanced technology. The same cannot be said for the functional changes. This is because function is difficult to access in the small and fragile cardiovascular system that is also highly sensitive to any kind of perturbation. So what we know about functional changes at these stages is proportionately small. The articles in this issue exhibit a range of creative approaches in investigating the function of the developing cardiovascular system that is highly influential to the development of the embryo and placenta. The wider use of these and other methods to assay function may open up a new age of discovery to explain the etiology of the molecular and structural changes of cardiovascular development.

The development of a functional cardiovascular system is well coordinated between formation of the beating heart with the development of the intra- and extra- embryonic vasculature and blood cells. It is the first organ system to develop during embryogenesis to enable the exchange of nutrients and gasses between the maternal blood and that of the developing fetus. A functional cardiovascular organ system is necessary for the growth and development of the embryo. As the heart begins to form from the lateral plate mesoderm, cardiomyocytes differentiate into contractile cells that quickly form a beating tubular structure. This straight heart tube initially displays a peristaltic wave of contraction to move the developing blood from the yolk sac vasculature through the cardiac tube and into the newly forming intra-embryonic blood vessels and subsequently away from the embryo again to the extra-embryonic vasculature of the yolk sac. As blood flow increases, an important process is initiated with an initial right-ward bend of the tube that ends with the formation of the four-chambered heart with the left and right atria being situated above the left and right ventricles with septa and valves developing between the chambers. This looping process must be orchestrated precisely or congenital cardiac defects can arise (Linask, 2003, 2013; Srivastava, 2006).

How the development of this dynamic organ-system is so precisely orchestrated is the focus of this Special Topic in Biophysics. It has become generally accepted that the forces associated with blood flow are important in helping to drive the looping process and the morphogenesis of the four-chambered heart and valves. It is a topic that cardiovascular developmental biologists, cell and molecular biologists, and bioengineers have analyzed from various angles now for more than 2 decades. This research topic brings together 10 articles from investigators in this field, both original and review articles, to provide an overview of the current status of the biophysical aspects of cardiovascular development and possible directions for future studies. In the 1990's the field relied mainly on experimental studies using avian embryos because the avian heart is easy to manipulate and to visualize under the microscope to see the changes occurring during heart looping (Taber et al., 1995). Effects of experimental manipulations on cardiac structure became evident from analyzing paraffin sections that were stained for specific protein markers as sarcomeric myosin heavy chain using MF20 antibody for the myocardium (Bader et al., 1982) or QH-1 antibody for the quail endocardium (Coffin and Poole, 1988). At that time technology was lacking to analyze both structure and heart function concomitantly and in real time. This changed with development of new biophotonic technological tools as optical coherence tomography. Karunamuni et al. (2014) describe in their review article “Capturing structure and function in an embryonic heart with biophotonic tools” the attributes of the technological advances in biophotonics and how it has helped to advance our knowledge of normal and abnormal cardiovascular development. Kowalski et al. (2014) in “Investigating developmental cardiovascular biomechanics and the origins of congenital heart defects” provides an overview of different approaches used to quantify embryonic cardiovascular functional maturation and the role of biomechanics in the regulation of cardiovascular morphogenesis and the role of computational modeling. It has become more recently recognized that changes in the hemodynamics of the extra-embryonic vitelline and placental blood flow contribute to changes in cardiac hemodynamics. Linask et al. (2014) focus on evidence from multiple studies in animal models as well as during human pregnancy on the impact of abnormal uteroplacental blood flow and changes in biophysical parameters that seemingly contribute to congenital heart defects. This theme is further discussed by Garcia and Larina (2014) in their review on “Vascular development and hemodynamic force in the mouse yolk sac” describing imaging methods and the molecular and biomechanical regulators guiding vascular remodeling. Next cardiac looping is discussed by two original research articles: Bayraktar and Manner (2014) in “Cardiac looping may be driven by compressive loads resulting from unequal growth of the heart and pericardial cavity. Observations on a physical simulation model” present data to test the physical plausibility of what they call the *growth-induced buckling* hypothesis using a simulation model. Shi et al. (2014) discuss their data on looping in the article “Bending and twisting the embryonic heart: a computational model for c-looping based on realistic geometry.” The article entitled “The impact of flow-induced forces on the morphogenesis of the outflow tract (OFT)” by Biechler et al. (2014) describes new original data from analyzing response of embryonic OFT tissue to different levels of fluid flow. The last three articles review our current understanding of biophysical attributes related to hemodynamics and mechanical signaling in “Mechanical regulation of cardiac development” by Lindsey et al. (2014). The effects of altering hemodynamics by surgical interventions are discussed in the article by Midgett and Rugonyi (2014). The field of extracellular matrix molecules and their role in mechanotransduction in heart development is represented by the review “Tenascin-C and mechanotransduction in the development and diseases of cardiovascular system” by Imanaka-Yoshida and Aoki (2014).

Together these articles provide an overview of the current understanding of biophysical aspects of the development of cardiovascular form and function. New data and new models are provided to explain important phases of heart development. As pointed out in the articles, always new questions are raised for future study.

## Conflict of interest statement

The Review Editor Ranganath Mamidi declares that, despite being affiliated to the same institution as the author Michiko Watanabe, the review process was handled objectively. The authors declare that the research was conducted in the absence of any commercial or financial relationships that could be construed as a potential conflict of interest.

## References

[B1] Bader D. Masakki T. Fischman D. A. (1982). Immunochemical analysis of myosin heavy chain during avian myogenesis *in vivo* and *in vitro*. J. Cell Biol. 95, 763–770. 10.1083/jcb.95.3.763 6185504 PMC2112936

[B2] Bayraktar M. Manner J. (2014). Cardiac looping may be driven by compressive loads resulting from unequal growth of the heart and pericardial cavity. Observations on a physical simulation model. Front. Physiol. 5:112. 10.3389/fphys.2014.00112 24772086 PMC3983514

[B3] Biechler S. V. Junor L. Evans A. N. Eberth J. F. Price R. L. Potts J. D.. (2014). The impact of flow-induced forces on the morphogenesis of the outflow tract. Front. Physiol. 5:225. 10.3389/fphys.2014.00225 24987377 PMC4060072

[B4] Coffin J. D. Poole T. J. (1988). Embryonic vascular development: immunohistochemical identification of the origin and subsequent morphogenesis of the major vessel primordia in quail embryos. Development 102, 735–748. 3048971 10.1242/dev.102.4.735

[B5] Garcia M. D. Larina I. V. (2014). Vascular development and hemodynamic force in the mouse yolk sac. Front. Physiol. 5:308. 10.3389/fphys.2014.00308 25191274 PMC4138559

[B6] Imanaka-Yoshida K. Aoki H. (2014). Tenascin-C and mechanotransduction in the development and diseases of cardiovascular system. Front. Physiol. 5:283. 10.3389/fphys.2014.00283 25120494 PMC4114189

[B7] Karunamuni G. H. Gu S. Ford M. R. Peterson L. M. Ma P. Wang Y. T.. (2014). Capturing structure and function in an embryonic heart with biophotonic tools. Front. Physiol. 5:351. 10.3389/fphys.2014.00351 25309451 PMC4173643

[B8] Kowalski W. J. Pekkan K. Tinney J. P. Keller B. B. (2014). Investigating developmental cardiovascular biomechanics and the origins of congenital heart defects. Front. Physiol. 5:408. 10.3389/fphys.2014.00408 25374544 PMC4204442

[B9] Linask K. K. (2003). Regulation of heart morphology: current molecular and cellular perspectives on the coordinated emergence of cardiac form and function. Birth Defects Res. C Embryo Today 69, 14–24. 10.1002/bdrc.10004 12768654

[B10] Linask K. K. (2013). The heart-placenta axis in the first month of pregnancy: induction and prevention of cardiovascular birth defects. J. Pregnancy 2013:320413. 10.1155/2013/320413 23691322 PMC3652177

[B11] Linask K. K. Han M. Bravo-Valenzuela N. J. M. (2014). Changes in vitelline and utero-placental hemodynamics: implications for cardiovascular development. Front. Physiol. 5:390. 10.3389/fphys.2014.00390 25426076 PMC4227466

[B12] Lindsey S. E. Butcher J. T. Yalcin H. C. (2014). Mechanical regulation of cardiac development. Front. Physiol. 5:318. 10.3389/fphys.2014.00318 25191277 PMC4140306

[B13] Midgett M. Rugonyi S. (2014). Congenital heart malformations induced by hemodynamic altering surgical interventions. Front. Physiol. 5:287. 10.3389/fphys.2014.00287 25136319 PMC4117980

[B14] Shi Y. Yao J. Young J. M. Fee J. A. Perucchio R. Taber L. A. (2014). Bending and twisting the embryonic heart: a computational model for c-looping based on realistic geometry. Front. Physiol. 5:297. 10.3389/fphys.2014.00297 25161623 PMC4129494

[B15] Srivastava D. (2006). Making or breaking the heart: from lineage determination to morphogenesis. Cell 126, 1037–1048. 16990131 10.1016/j.cell.2006.09.003

[B16] Taber L. A. Lin I. E. Clark E. (1995). Mechanics of cardiac looping. Dev. Dynamics 203, 42–50. 10.1002/aja.1002030105 7647373

